# “*You have a self-testing method that preserves privacy so how come you cannot give us treatment that does too?”* Exploring the reasoning among young people about linkage to prevention, care and treatment after HIV self-testing in Southern Malawi

**DOI:** 10.1186/s12879-022-07231-7

**Published:** 2022-04-21

**Authors:** Lisa Harrison, Moses Kumwenda, Lot Nyirenda, Richard Chilongosi, Elizabeth Corbett, Karin Hatzold, Cheryl Johnson, Musonda Simwinga, Nicola Desmond, Miriam Taegtmeyer

**Affiliations:** 1 Department of International Public Health, Liverpool School of Tropical Medicine, Liverpool, UK; 2 Malawi Liverpool Wellcome Trust Clinical Research Programme, Blantyre, Malawi; 3 School of Public Health and Family Medicine, College of Medicine, University of Malawi, Blantyre, Malawi; 4 Department of HIV Prevention, Population Services International, Blantyre, Malawi; 5 Department of Clinical Research, London School of Hygiene and Tropical Medicine, London, UK; 6 Population Services International, Johannesburg, South Africa; 7 Department of Global HIV, Hepatitis and STIs Programmes, World Health Organization, Geneva, Switzerland; 8 Zambia AIDS Related Tuberculosis Project, Lusaka, Zambia; 9 Clinical Sciences Department, Liverpool School of Tropical Medicine, Liverpool, UK; 10 Tropical Infectious Diseases Unit, Royal Liverpool University Hospital, Liverpool, UK

**Keywords:** Adolescents, Young people, HIV self-testing, Malawi, Linkage, Community-based health

## Abstract

**Background:**

Young people, aged 16–24, in southern Malawi have high uptake of HIV self-testing (HIVST) but low rates of linking to services following HIVST, especially in comparison, to older generations. The study aim is to explore the barriers and facilitators to linkage for HIV prevention and care following uptake of HIV self-testing among young Malawians.

**Methods:**

We used qualitative methods. Young people aged 16–24 who had received HIVST; community-based distribution agents (CBDAs) and health care workers from the linked facilities were purposively sampled from two villages in rural southern Malawi.

**Results:**

We conducted in-depth interviews with thirteen young people (9 female) and held four focus groups with 28 healthcare workers and CBDAs. Young people strongly felt the social consequences associated with inadvertent disclosure of HIV sero-status were a significant deterrent to linkage at their stage in life. They also felt communication on testing benefits and the referral process after testing was poor. In contrast, they valued encouragement from those they trusted, other’s positive treatment experiences and having a “strength of mind”. CBDAs were important facilitators for young people as they are able to foster a trusting relationship and had more understanding of the factors which prevented young people from linking following HIVST than the healthcare workers. Young people noted contextual barriers to linkage, for example, being seen on the road to the healthcare centre, but also societal gendered barriers. For example, young females and younger adolescents were less likely to have the financial independence to link to services whilst young males (aged 19–24) had the finances but lacked a supportive network to encourage linkage following testing. Overall, it was felt that the primary “responsibility” for linking to formal healthcare following self-testing is shouldered by the young person and not the healthcare system.

**Conclusions:**

Young people are happy to self-test for HIV but faced barriers to link to services following a self-test. Potential interventions for improving linkage suggested by this analysis include the establishment of youth-friendly linkage services, enhanced lines of communication between young people and healthcare providers and prioritising linkage for future interventions when targeting young people following HIVST.

## Background

In the past decade, public health strategies in Sub-Sahara Africa (SSA) have focused on increasing young people’s access to HIV testing and treatment services because of the high HIV incidence and low uptake of testing in this sub-group [1, 2]. Adolescence is widely recognised as a period of physical and mental development, a time of testing boundaries, increasing independence and risk-taking, which elevates their vulnerability to HIV infection [3–5].

Malawi has a young and growing population. In 2018, a fifth of the population was aged 15–24 years and nearly 32% of new HIV infections occurred in this group and a majority among female adolescents [6–8]. Young people in Southern Malawi have an increased vulnerability to HIV as they are less likely to use condoms and more likely engage in sexual activity at a younger age [8]. Additionally, pre-marital sex is strongly disapproved of which creates a “culture of silence” whereby the negative consequences of sex are emphasised so young people fear discussing sexual health issues with their parents or peers [9]. Myths and misconceptions about HIV transmission and treatment spread among adolescents and further elevate HIV risk [10]. There are multiple reasons why young women and girls in Malawi experience this high HIV burden including being more likely to experience sexual violence, intergenerational relationships and experiencing a younger age of sexual debut and marriage than their male counterparts [9]. Furthermore, adolescents in Malawi experience lower HIV testing coverage then their adult counterparts, exemplified in a 2015–16 survey where 79% of adults (over 24 years) had ever received HIV testing and results in comparison to only 40.4% of adolescents (15–19 years old) [11].

Reaching young people with HIV testing and prevention and care services is a priority of the Malawi HIV and AIDS Strategy [12]. HIV self-testing at home has played a key role in increasing testing uptake in this group [1, 13]. Choko et al. (2015) found that adolescents in Malawi, had the highest uptake of HIVST over a 2-year period, due to increased confidentiality, convenience and ease of use and 56% of users, of all ages, linked to treatment [13]. As a result of this data, HIVST for young people became a key focus in Malawi for the HIV Self-Testing Africa (STAR) Initiative with implementation led by Population Services International (PSI) a locally based international NGO working on the ground through community-based distribution agents (CBDAs) on short term contracts, who distribute the HIVST kits to people of all ages in the community. Whilst distributing these tests the CBDA’s advise and give information to all users on how to link to formal health services for confirmation testing, however they are not trained in professional post-test counselling. Included in the HIVST kit is a referral card which all users present at the formal health services.

Despite the high acceptance of HIVST, young people in Malawi are less likely to link to formal health services post-HIVST [14, 15]. This creates a treatment gap between the adult and younger population evidenced in 2016 where 41% of HIV positive adolescent and young Malawian people aged 15–24 were aware of their status and receiving Antiretroviral Therapy (ART) in comparison to of 69% adults [11]. Reported barriers for young people to accessing general sexual and reproductive health (SRH) services in Malawi include; fear of HIV-related stigma and discrimination, the lack of confidential and “youth-friendly’ SRH services and the associated cost of transport [16–21]. Reportedly, receiving STI treatment and therefore keeping their STI status private is a motivation for young Malawian people to access sexual health services as this preserves their social status in the community [17], the same hasn’t yet been revealed, however, for young people’s reasoning in linking to HIV services. The U = U (Undetectable = Untransmittable) campaign is a powerful message for people living with HIV (PLHIV) to encourage linkage to post-testing services and adherence to treatment [22]. Yet, young people in Malawi may not have knowledge of this campaign as in 2016, just 41% of women and 44% men (aged 15–24) had a “comprehensive knowledge of HIV”—which did not include knowledge of the U = U campaign [8].

We investigated self-tested young people’s perceptions on the barriers and facilitators to linking to services after HIVST.

## Methods

A cross-sectional, inductive, qualitative approach was utilised to examine young people’s perceptions about the reasons for linking, or not linking, to confirmatory testing and treatment after receiving a positive self-screen result.

### Study setting

From the most recent national demographic health survey in 2015–16, the southern region of Malawi has the nationally highest HIV prevalence [8]. Two villages within the Southern Machinga district were chosen for sampling locations as unpublished process data from STAR (2018) demonstrated that young people who self-tested from the Southern Machinga district had comparatively low rates of linkage to services. Village 1 was purposively chosen to sample participants because it had the lowest rates of linkage in the district and, therefore, likely held the richest data. Village 2 in the same district was chosen for comparison as it was the closest village with higher rates of linkage to Village 1.

### Sampling strategy

To approximate information saturation in the short time frame provided, rapid appraisal methods were used with swift participation sampling rather than other naturalistic methods [23, 24]. Young people were sampled purposively, where predicted information-rich participants were recruited by local CBDAs, who would verbally describe the study so the young people could provide informed consent. Young people aged 16–24 were sampled, with a deliberate majority of females and efforts to include younger adolescents (below 18 years old). To avoid stigma associated with study-participation, participants were not required to reveal their HIV status or whether they linked to services post-HIVST.

Healthcare workers and CBDAs were sampled from the village’s health centres, balanced in age and gender. Included healthcare providers were of the same cadre to lessen professional hierarchy bias.

### Data collection

Rapid appraisal methods; such as in-depth interviews (IDI) with purposive opportunistically sampled participants and focus group discussions (FGD) of key informants, aim to assess and address a community’s health needs within a limited time frame to give practical recommendations and was therefore chosen to approximate information saturation in the short time frame provided [24].

In-depth interviews were adopted with the 16–24-year-old study participants as these allowed for an in-depth exploration of their individual perspectives in relation to their underpinning characteristics and beliefs [23]. Furthermore, as this involved discussion of sensitive topics like HIV testing, a focus group discussion would likely inhibit responses about personal behaviour due to the lack of confidentiality. FGDs was chosen to explore the collective perspectives of the HIVST suppliers; healthcare workers and CBDAs (in separate FGD’s), to additionally observe any conflicts or agreements between the villages and professions [25].

IDIs and FGDs were held in the participants’ preferred languages of Chichewa or Chiyao by a research assistant (male, aged 25) who had previously lived and worked in the area to further build trust with the participant but also impart contextual knowledge to the main researcher (LH, female aged 26), who took observational notes and made further iterative enquiries to further explore previously unexpected topic areas. A semi-structured interview topic guide was used to guide discussions, with open questions such as “What are your opinions of local health facilities and how they treat young people?” and further solution-orientated questions, for instance “if you could organise how young people link to services after HIVST, what would you do?”. The topic guide also included methods of free-listing, ranking and scenarios as described by Palmer [24]. Free listing involved the participants listing any reasons young people would link to services (or not), these were then ranked by perceived importance to give means of comparison. These reasons and rankings were referenced for further exploration within the interviews. All participants were presented with hypothetical scenarios and asked to describe what would happen in their community, for example, “What would a young person do if they went to a health facility to get treatment and they saw someone else their age?”; “Would it be different if they knew them?”. This methodological triangulation helped build rapport, facilitated further exploration and safeguarded the data’s trustworthiness [26, 27].

### Data analysis

A thematic framework approach was used to analyse the qualitative data and observe differences between participants’ perspectives. After transcription and translation of data the lead researcher (LH) first familiarised and immersed herself in the data. From this immersion, a coding framework was developed using a priori/deductive codes and each IDI and FGD was double-coded after the transcripts had been imported in NVivo 11 (QSR-Australia). Categories of coded data were grouped to reveal overarching themes. Participant’s responses were compared between villages, young people, healthcare workers and CBDAs. Data triangulation was also stratified by the socio-demographic characteristics of; age, sex, village and HIV status where the latter had been disclosed voluntarily.

Ethical approval for this project was given by the Liverpool School of Tropical Medicine (LSTM) on the 29th March 2018 and in Malawi by the College of Medicine Research and Ethics Committee (COMREC, Protocol number:P.01/16/1861) on the 13th April 2018 under research with the STAR (HIV Self-Testing Africa Initiative) affiliated with Malawi-Liverpool Wellcome Trust (MLW).

## Results

We included 41 participants (see Table 1). Thirteen IDI’s were conducted with the young participants and an additional 28 individuals participated in four FGD’s with two groups of healthcare workers and two groups of CBDAs from each village. Although the young study participants were not required to reveal their HIV status, 12 out of 13 participants voluntarily disclosed this during the IDIs. By chance, most young people sampled from Village 1 had a HIV + status whereas as the majority from Village 2 had a HIV − status.

**Table 1:** Participants demographic characteristics

Categories/sub categories	Data collection approach		
	In-depth Interviews (n = 13)	Focus group discussions (n = 28)	
Population group	Young HIV self-testers	Health Care Workers (16)	Community Based Distributing Agents (12)
Location			
Village 1	7	7	Village 1 (6)
Village 2	6	9	Village 2 (6)
Sex			
Female	9	8	8
Male	4	8	4
Age			
16–19	3	N/A	N/A
20–24	10	0	2
25–30		8	3
30 +		8	7
HIV status			
HIV-Positive	7	N/A	N/A
HIV-Negative	6		
Unknown	1		
Totals	13	16	12

Confidentiality and not wanting to disclose HIV status, social support, communication, and attitudes/perceptions toward the health facilities emerged as key themes on factors influencing linkage to healthcare following a positive HIVST result at home for young people.

### The contextual impact on the assurance of confidentiality

Most participants agreed that young people would not link to formal health services following a positive HIVST result due to the anticipated negative social consequences of inadvertent disclosure of their HIV status. Interestingly and in contrast to the majority, three young people (2 female and 1 male, aged 23–24) from Village 1 described how early linkage to confirmatory testing and subsequent treatment preserved their serostatus privacy;

*“You try to hide it from people because when you wait for you to be sick people get to realize what is really wrong with you” (Male, 23)*

A healthcare provider from Village 1 described that the facility held youth-specific days for HIV services supported young people’s access to post-HIVST services due to the social support from their peers and youth-friendly health providers. Four of the seven young people and CBDAs from Village 1, however, narrated that this approach as an obstacle as it is not effective in safeguarding their privacy in their serostatus or that they were accessing post-HIVST services, and preferred using healthcare centres further away but with less visible routes;

*“When people in the village see [young people] going on that day, they definitely judge they are going for treatment and mock them” (Female, 23)*

In conflict with this position, nearly all young people ranked the long distance to healthcare centres and associated transport costs as a highly important barrier to linkage. One CBDAHIVST provider from Village 1 described young people’s frustration on the lack of a convenient and private approach for linking to services for those self-testing HIV positive;

*“[Young people] tell us that ‘you have a self-testing method that preserves privacy so how come you cannot give us treatment that does too?’” (Village 1 CBDA FGD Participant)*

### Availability of social support and trusting relationships

The presence or absence of social support from a trusted individual was described as an important factor for youth to access confirmatory testing and treatment following HIVST. There were conflicting views on parental support; three young females (aged 16–22) across the villages’ stated parents were supportive as they are “wise” whereas three young people in Village 2 (two males and one female aged 18–23) expressed concerns that their parents may compromise their unwillingness to disclose their serostatus publicly.

Eleven young people, both CBDA and the village 2 healthcare FGD participants, observed that CBDAs were well placed to foster trusting relationships with young people and could encourage accessing formal health services following HIVST because they lived and worked in the community and could be accessed in the village for advice without young people experiencing fear of inadvertent HIV status disclosure;

*“Even when [my friend] has some health problems, he consults the CBDA, I even saw him yesterday going to the CBDAs house” (Male, 20).*

Despite their placement in the community, CBDA’s were perceived as confidential figures as the young people had observed them talking to their peers and their peer’s serostatus was not revealed within the community.

Four young people (two male and two females, aged 18–23) described peer support as a facilitator to linkage following HIVST but these were often informal and with no mention of sexual partners. For example, one young self-tester from Village 1 (Female, 21) narrated that some young PLHIV created a secret support group to encourage each other’s treatment adherence. However, many young people and participants in the four FGDs discussed how the general perception that HIV treatment is not a cure leads to a fatalistic attitude even among peers;

*“[Young people] don’t go because of peer influence, when they disclose to their friends, [they] influence them to not to go to the hospital… Because they say the virus has no cure (Female, 24)”*

Both village healthcare centres were said to have “expert client” volunteers in the facilities who were presented to patients as “treatment role models”. The healthcare workers and some CBDAs were convinced that expert clients had an important role to play in facilitating health-service linkage and treatment adherence among HIV self-tested youth;

*“When youths see these HIV positive people living happy and healthy, they get encouraged and see no reason of isolating themselves from treatment.” (Village 1 healthcare worker FGD)*

In contrast, “expert clients” were ranked of low importance by the young people, and were even described as “ineffective” by a healthcare worker in Village 2, who narrated that no expert client is under 25-years-old and their ‘*counselling’* is only confined to the health facility.

### Communication between CBDAs, healthcare workers and NGO staff

The most notable difference between the villages was the relationship between healthcare workers, CBDAs and NGO staff. In Village 2, this relationship was a facilitator, but a barrier in Village 1. A healthcare worker from the Village 1 health centre lamented that the CBDAs didn’t counsel young people when they received a positive result, even though CBDAs are trained in HIVST distribution, not professional post-test counselling. This suggests that the CBDAs’ role was not fully explained to the healthcare workers in Village 1. A young woman from Village 1 described how the fractured relationship between CBDAs and healthcare workers hindered her access to post-HIVST services when she presented at the facility;

*“[The healthcare workers] didn’t believe that I have done self-testing, [my CBDA] gave me a referral card, I said I don’t see any reason to not believe me.” (7FYP, 21)*

The healthcare workers in Village 2 described how they experienced a similar scenario and the NGO organised a meeting to explain the roles of the professionals to each other and confirmed referral methods. In this meeting, it was decided that mobile phones would be used to support referrals of young HIV self-testers between CBDAs and healthcare workers based at a facility. However, a meeting of this nature had not taken place in Village 1.

Participants in all the four FGDs desired more interaction and communication between CBDAs and health workers to ensure seamless referrals and to prevent future tension, as articulated;

*“We were not involved in any of the meetings which the project implementers conducted with the CBDAs, so we had no clue on what role we were playing and what role were the CBDAs playing.” (Village 1 Healthcare worker FGD Participant)*

Furthermore, this lack of communication between the healthcare providers, CBDAs and self-tested young people contributes to a failure in recognising young people’s barriers to post-HIVST services linkage. The young people and CBDAs ranked the following four factors of high priority for linkage (special treatment days, distance as barriers and encouraging CBDAs and “strength of mind” as facilitators) whereas the healthcare workers ranked these factors as low priority.

### Role of health centres in promoting linkage

Seven young people and all of the FGD participants ranked healthcare services as the most important facilitator to ensure linkage to confirmatory testing and HIV treatment and care. The need for a confirmatory test was said to encourage young people to link in order to dispel anxieties on whether young self-testers performed the self-test accurately. Furthermore, eight young people stated they, and other peers, linked to health centres after observing HIV symptoms and to “stay healthy”. No young person mentioned the U = U campaign as an incentive for linkage, however, this was also not probed for.

The FGD participants described the healthcare workers providing confidential counselling and treatment as another facilitator. Most young people described healthcare workers as respectful or “*welcomed them properly*”. Noticeably, young people and healthcare workers both described this respect as reciprocal;

*“It depends on the attitude of the [young] person …. We don’t shout at them but on the issue of respect it depends on their attitude (Laughter) (Pause) According to our job and hospital rules we still respect [by not shouting or getting angry with] them whether they respect us or not” (Village 2 Healthcare worker FGD Participant)*

### Influence of demographic characteristics on linkage

When the perspectives were stratified by gender, it was observed that young males were more concerned with the impact on their societal status in the community or loss of sexual partners from a potential inadvertent HIV status disclosure during linkage. In contrast, young females were afraid of the social consequences in terms of losing potential future marriage proposals and financial security. Additionally, young women described how they were less able to afford any transport costs as they held less lucrative jobs living with their parents;

*“A boy would emigrate and work at tobacco farms to buy a bike while a girl wouldn’t” (Female, 23)*

More females stated having “strength of mind” as an important facilitator. This may relate to a CBDA’s observation in Village 1 that females are more likely to have a close relationship with their parents at home, disclose their status and receive encouragement to have the ‘strength of mind’ to link to formal health services. For the young men, the story was different as illustrated in a quote below;

*“It is really difficult for young people, mostly boys, they take time to disclose their results to their parents, they are hard to convince them to accept it, it is like you’re working with a lion” (Village 1 CBDA FGD Participant)*

In terms of age, study participants falling within the 16–19 and 20–24-year-old age groups held similar perspectives on linking (or not) to post-HIVST services. However, this is likely due to the lack of comparative data as only three participants were aged 16–19, and they seemed to find it difficult to fully articulate their reasoning for linking to services or not. One young person from Village 2 (aged 23) described how adolescents find the associated travel costs a larger obstacle than their older counterparts as they are more likely to be financially dependent on their parents. Additionally, a healthcare worker noted how the cultural perceptions of adolescents contribute towards undermining agency to make independent decisions;

*“According to our Malawian culture, a person who is sixteen is still a little child…, he needs a guardian to guide him” (Village 1 Healthcare worker FGD Participant)*

The village of residence had a bearing on the ability to link to services following HIVST. A major difference between the villages was that young people from Village 2 described living a “sexually-risky” lifestyle as a driver to link to post-test confirmatory testing and treatment services as illustrated in this quote;

*“Most male young people who go to the lake earn huge amounts of money which influence them to indulge in sexual relationships with all kinds of people and when they test themselves Negative that’s when they come for verification” (Village 2 CBDA FGD Participant).*

This “sexually-risky” lifestyle is likely related to the available income from fishing activities in Village 2, and not in Village 1, so young men from this village were more likely to engage in transactional sex. Furthermore, more young people in Village 1 discussed issues relating to travel “visibility” as there is a single route to the nearest health-centre which the local community lives alongside. Meanwhile in Village 2, there are multiple, less visible routes to the health facility.

## Discussion

This study is one of few to describe young people’s perceptions of barriers and facilitators to linking to services following HIVST. Similarly to Malawi adults and echoing previous findings, confidentiality and the self-preservation of their serostatus pervaded young people’s reasoning for linking to post-HIVST services [18, 28]. This was gendered and affected by young people’s age, reiterating Hatchet et al. (2012) [29], as adolescents and young males were less likely to disclose their status to others and so experience less support in linking to post-HIVST services. Our finding that young people emphasized having “strength of mind” as a facilitator highlights how young people feel expected to shoulder the burden of linking to formal healthcare services by overcoming a multitude of barriers*.*

An unexpected barrier was that of poor communication/relationship between CBDAs working in the community distributing HIVST kits and the healthcare workers based at a clinic who provide follow-up services. This finding shows a “fragmented” approach to implementation, with a focus on increasing HIV testing uptake but little emphasis on subsequent linkage to post-HIVST services like confirmatory testing or treatment. This lack of joined up thinking can negatively impact sexual health outcomes [30]. Community health programmes that effectively work with close-to-community providers have been shown to successfully reach pregnant adolescents or young people who were lost to follow-up from HIV care, by building on the important interface role that close to community providers have between the health system and the communities they serve [31, 32]. Deliberately aligning tasks to national community-health programmes is likely to ensure improved community support to post-test linkage in adolescents during a period when international funding for bespoke support is declining [31–33]. Our findings on the importance of the CBDA cadres give further evidence how it is vital to understand the local context to address issues of professional distrust or unfamiliarity which affect upon service delivery performance and impact [31, 32].

Youth participation should be a programme priority to increase young people’s access and use of SRH services [34–36]. However, this study showed a lack of communication between the young people and the healthcare workers. In Kenya, a study reported that having stakeholders and a young person’s “advisory group” involved in the planning of treatment distribution, improved linkage by 41% [37]. Therefore, this should be a priority in future HIVST implementation to increase young people’s linkage to post-HIVST health services.

As with previous findings, [18, 38], this study also highlighted how CBDAs were motivators and preferred by young people because they were trusted and lived in the villages with the young people, whereas this trust in healthcare professionals was only experienced by those who linked to services.

In this study, ‘expert clients’ roles were described as confined within the health facilities with a limited role in the community. This is a lost opportunity, as multiple studies [21, 29, 39] have reported how integrating peer supporters, especially PLHIV, into the healthcare system during follow-up visits can be especially effective in increasing young people’s linkage. Hence, they could effectively promote linkage for the young people by working in conjunction with the CBDAs in the community. Expert clients and/or CBDAs could also provide a means of transport which has also been found to increase linkage for young people [40, 41] and help to improve communication between those in the community and healthcare providers based at the referral facility.

### Methodological limitations

Using CBDA recommendations for purposive sampling may have created a sample bias towards the young people’s positive CBDA descriptions as the CBDAs likely chose participants with whom they had a positive relationship. Furthermore, there was an unintended location bias whereby most young people sampled from Village 1 disclosed a HIV + status and in Village 2 a HIV − status. All the young PLHIV had linked to post-HIVST services and as such their perspectives and experiences may have been different from those of the individuals who failed to link. Most of the HIV negative young people’s perspectives were based on other’s experiences or hypothetical thinking. This lack of comparison with experiential data was mitigated through the triangulation of data from different sources and the range of participants provided a holistic view of linkage reasoning from the HIVST supply and demand perspectives for young people.

Despite the sensitive nature of the discussions with the young people, the trust gained with the researchers in the limited time frame is evidenced through the majority of participants self-disclosure of their serostatus. This may be due to the researchers being balanced in gender and of a relatively younger age, 25 and 26.

## Conclusions

In conclusion, HIVST provides confidential and convenient testing which increases uptake among young people. However, linking to confirmatory testing and treatment following a self-test at home remains a daunting challenge as the onus of responsibility to link rests on the young people and their ability to navigate multiple and complex barriers. This study shows the importance of communication between target beneficiaries and the different health providers involved within a health intervention delivered at community level. It also highlights young people’s reasoning for linking to services post-HIVST, including the differing contextual and gendered perspectives, which future Malawian policy makers and implementers can use to implement effective interventions with a targeted response to encouraging different groups of young people’s linkage post-testing. Implementing these recommendations in the national scale-up of HIVST in Malawi would maximise the benefits of young people’s high uptake of HIVST; so more young people receive treatment to achieve viral suppression and, ultimately, reduce the national incidence and prevalence of HIV in Malawi.

## Data Availability

The datasets used and/or analysed during the current study are available from the corresponding author on reasonable request.

## Abbreviations

ART: Antiretroviral therapy
CBDAs: Community-based distributing agents
FGD: Focus group discussions
HIVST: HIV self-testing
IDI: In-depth interviews
MLW: Malawi-Liverpool Wellcome trust
PLHIV: People living with HIV
SSA: Sub-Saharan Africa
STAR: HIV self-testing Africa Initiative
WHO: World Health Organisation
